# Supplementary material for: Neutralizing activity of BBIBP-CorV vaccine-elicited sera against Beta, Delta and other SARS-CoV-2 variants of concern
Source: Nat Commun. 2022 Apr 4;13:1788. doi: 10.1038/s41467-022-29477-0 (PMC8980020; doi:10.1038/s41467-022-29477-0)
Supplement: Supplementary file 1 — Supplementary Information [file 41467_2022_29477_MOESM1_ESM.pdf]

Supplementary Information

Neutralizing activity of BBIBP-CorV vaccine-elicited sera against Beta, Delta and other SARS-CoV-2 variants of concern

Supplementary Table 1. Baseline demographic characteristics of participants at each time point tested for neutralizing activity.

	V3	V4		V5	
	Wild-type (n=964)	Wild-type (n=760)	Variants (n=470)	Wild-type (n=581)	Variant (n=220)
Age	35.00 (28.00-43.00)	36.00 (30.00-43.00)	35.00 (30.00-43.00)	37.00 (30.00-44.00)	36.00 (30.00-44.00)
Sex					
Male	283 (29.36%)	203 (26.71%)	121 (25.74%)	138 (23.75%)	52 (23.64%)
Female	681 (70.64%)	557 (73.29%)	349 (74.26%)	443 (76.25%)	168 (76.36%)

Data are expressed as median (interquartile range [IQR]) or number (%).

V3: on day 21 after the first dose; V4: on day 28 after the second dose; V5: on day 180 after the second dose.

Multiple variants tested at V4 include variants Alpha, Beta, Gamma, and Iota.

Variant tested at V5 includes variant Delta.

Supplementary Methods: Study protocol

This study is registered with ClinicalTrials.gov Identifier: NCT04795414.

1. Protocol Summary

1.1 Study title

Safety and immunogenicity of an inactivated vaccine against COVID-19 in medical workers

1.2 Brief Summary:

With the unprecedented morbidity of the COVID-19 pandemic, the vaccine effectiveness needs to be assessed across diverse populations. The purpose of this study is to evaluate the safety and immunogenicity of an inactivated SARS-CoV-2 vaccine in medical workers.

1.3 Detailed Description:

1370 participants who are negative for serum-specific antibodies against SARS-CoV-2 at the time of screening are enrolled and will receive a two-dose schedule, 21 days apart, with 4 μg BBIBP-CorV inactivated SARS-CoV-2 vaccine. The primary safety endpoint includes solicited local and systemic reactions prompted by and recorded in an electronic diary within 7 days post each injection, unsolicited adverse events and serious adverse events assessed from the receipt of each dose. The second safety endpoint is the clinical laboratory abnormalities from dose 1 through 1 month after dose 2, which including measurement of alanine aminotransferase, aspartate aminotransferase, serum total bilirubin, serum albumin, creatinine, blood urea nitrogen, and blood routine examination. The primary immunogenic endpoint is assessed as the serum anti-SARS-CoV-2 specific antibody responses and neutralizing activity at 4 weeks after the second vaccination.

2. Study Design

Study type: observational

Estimated enrollment: 1370 participants

Observational model: cohort

Time perspective: prospective

Actual study start date: January 14, 2021

Estimated primary completion date: April 30, 2022

Estimated study completion date: January 31, 2023

3. Study intervention

3.1 Group

Vaccination Group

Participants vaccinated with inactivated SARS-CoV-2 vaccine are studied for safety and antibody response.

3.2 Intervention

Biological: SARS-CoV-2 Vaccine (Vero Cell), Inactivated

The subjects of the Vaccination Group will receive two doses, 21 days apart of inactivated SARS-CoV-2 vaccine (4 µg of BBIBP-CorV)

4. Study Population

Eligibility Criteria

Medical workers in Ruijin Hospital who are willing to receive two doses of SARS-CoV-2 inactivated vaccine via intramuscular injection in the arm.

4.1 Inclusion Criteria:

- Written informed consent according to ICH/GCP regulations prior to any trial specific procedures.
- Male or female, aged 18-59 years.

4.2 Exclusion Criteria:

- Allergy to any ingredient included in the vaccine;
- A history of seizures or mental illness;
- Individuals with any acute febrile disease on the day of vaccination;
- Women who are pregnant or breastfeeding.

4.3 Participant discontinuation/withdrawal

A participant may withdraw from the study at any time at his/her own request. Reasons for discontinuation from the study may include:

- Refused further follow-up;
- Lost to follow-up;
- AEs;
- Participant request.

4.3 Sample collection

Biospecimen Retention: Samples With DNA.

Blood samples (8-10 mL), including plasma, serum, and blood cell.

5. Follow-up visit description

5.1 Screening stage

V1, D0

The subjects whose basic information meet the criteria of the study are recruited for screening. The subjects must sign the informed consent. The information to be collected for screening include but not limited to:

Demographic information (gender, age, nationality), medical history, nucleic acid screening for SARS-CoV-2.

5.2 Inoculation stage

V2, D1

The eligible subjects will have a physical examination. Before vaccination, blood samples will be collected for blood routine and blood chemistry examination, humoral immunogenicity analysis, and inflammatory cytokines measurement. Then the first dose of vaccine will be inoculated. There will be a 30 minutes close observation after inoculation. Solicited local and systemic reactions will be prompted by and recorded in an electronic diary post injection. Unsolicited adverse events and serious adverse events will be assessed from the receipt.

V3, V2+21

The subjects will have a physical examination. Before vaccination, blood samples will be collected for blood routine and blood chemistry examination, humoral immunogenicity analysis, and inflammatory cytokines measurement. Then the second dose of vaccine will be inoculated. There will be a 30 minutes close observation after inoculation. Solicited local and systemic

reactions will be prompted by and recorded in an electronic diary post injection. Unsolicited adverse events and serious adverse events will be assessed from the receipt.

5.3 Follow-up stage

V4, V3+28

The subjects will have a physical examination. Blood samples will be collected for blood routine and blood chemistry examination, humoral immunogenicity analysis, and inflammatory cytokines measurement. Solicited local and systemic reactions will be prompted by and recorded in an electronic diary within 7 days post each injection. Unsolicited adverse events and serious adverse events will be assessed from the receipt.

V5, V3+180

Blood samples will be collected for humoral immunogenicity analysis and inflammatory cytokines measurement. Unsolicited adverse events and serious adverse events will be assessed from the receipt.

V6, V3+365

Blood samples will be collected for humoral immunogenicity analysis and inflammatory cytokines measurement. Unsolicited adverse events and serious adverse events will be assessed from the receipt.

6. Outcome Measures

6.1 Primary Outcome Measures

1. Percentage of participants reporting local reactions [Time Frame: within 7days post each vaccination]

   Symptoms including: Pain at the injection site, swelling, itch, and redness.

2. Percentage of participants reporting systemic events [Time Frame: within 7days post each vaccination]

   Symptoms including: Fever, headache, fatigue, nausea, vomiting, diarrhea, constipation, myalgia, arthralgia.

3. Percentage of participants reporting adverse events [Time Frame: From Dose 1 through 1 month after Dose 2]

   Adverse events are verified by the investigators.

4. Percentage of participants reporting serious adverse events [Time Frame: From Dose 1 through 1 month after Dose 2]

   Serious adverse events are verified by the investigators.

5. The seroconversion rate and the titres of serum anti-SARS-CoV-2 specific antibody and neutralizing antibodies against SARS-CoV-2 [Time Frame: at 1 month after dose 2]

   Determinate the levels of anti-SARS-CoV-2 specific antibody and the geometric mean titres (GMTs) of neutralizing antibodies against SARS-CoV-2.

6.2 Secondary Outcome Measures

1. Clinical laboratory abnormalities [Time Frame: From Dose 1 through 1 month after Dose 2]

   Laboratory tests included measurement of alanine aminotransferase, aspartate aminotransferase, serum total bilirubin, serum albumin, creatinine, blood urea nitrogen, and blood routine examination.

2. The seroconversion rate and the titres of serum anti-SARS-CoV-2 specific antibody and neutralizing antibodies against SARS-CoV-2 [Time Frame: at 6 months after dose 2]

   Determinate the levels of anti-SARS-CoV-2 specific antibody and the geometric mean titres (GMTs) of neutralizing antibodies against SARS-CoV-2.

3. The seroconversion rate and the titres of serum anti-SARS-CoV-2 specific antibody and neutralizing antibodies against SARS-CoV-2 [Time Frame: at 12 months after dose 2]

   Determinate the levels of anti-SARS-CoV-2 specific antibody and the geometric mean titres (GMTs) of neutralizing antibodies against SARS-CoV-2.

7 Statistical considerations

7.1 Sample size determination

The sample size was not determined based on the statistical power calculation. When 1370 participants are enrolled, we have 80% power to detect a higher rate than 98% when assuming the true rate is 99% in immunogenicity, with a 10% dropout rate at a two-sided significance level of 0.05 using a binomial test for one proportion in PASS13.

7.2 Analysis set

The safety analysis is based on the safety set consisting of participants who received at least one dose of vaccine. Humoral immunogenicity analysis is based on the per-protocol set consisting of enrolled participants who had received two doses of vaccine with blood sample collection after each vaccination.

7.3 Statistical analysis

Continuous variables with normal distribution will be presented as mean ± standard deviation [SD], and continuous variables that are not normally distributed will be presented as median (interquartile range [IQR]). Categorical variables will be described as count (%). The statistical method used for analysis of antibody titres is the geometric mean (GMTs) and the corresponding 95% confidence interval (95% CI). The values will be compared by Student's t test, the Wilcoxon signed-rank test, of the Mann-Whitney U test as appropriate. To assess the distribution in different groups, a chi-square test (□2) will be applied. Graphs will be plotted using GraphPad Prism 8.4.0. Statistical analyses will be done using SPSS 24.0. A two-sided *P* value of less than 0.05 is considered statistically significant.
