# Supplementary material for: Neutralizing activity of BBIBP-CorV vaccine-elicited sera against Beta, Delta and other SARS-CoV-2 variants of concern
Source: Nat Commun. 2022 Apr 4;13:1788. doi: 10.1038/s41467-022-29477-0 (PMC8980020; doi:10.1038/s41467-022-29477-0)
Supplement: Supplementary file 3 — Reporting Summary [file 41467_2022_29477_MOESM3_ESM.pdf]

Corresponding author(s): Xinxin Zhang, Jieming Qu, Zhitao Yang, Erzhen Chen

Last updated by author(s): Feb 24, 2022

Reporting Summary

Nature Research wishes to improve the reproducibility of the work that we publish. This form provides structure for consistency and transparency in reporting. For further information on Nature Research policies, see our Editorial Policies and the Editorial Policy Checklist.

Statistics

For all statistical analyses, confirm that the following items are present in the figure legend, table legend, main text, or Methods section.

n/a	Confirmed	
☐	☒	The exact sample size (n) for each experimental group/condition, given as a discrete number and unit of measurement
☐	☒	A statement on whether measurements were taken from distinct samples or whether the same sample was measured repeatedly
☐	☒	The statistical test(s) used AND whether they are one- or two-sided<br>*Only common tests should be described solely by name; describe more complex techniques in the Methods section.*
☒	☐	A description of all covariates tested
☒	☐	A description of any assumptions or corrections, such as tests of normality and adjustment for multiple comparisons
☐	☒	A full description of the statistical parameters including central tendency (e.g. means) or other basic estimates (e.g. regression coefficient) AND variation (e.g. standard deviation) or associated estimates of uncertainty (e.g. confidence intervals)
☐	☒	For null hypothesis testing, the test statistic (e.g. F, t, r) with confidence intervals, effect sizes, degrees of freedom and P value noted<br>*Give P values as exact values whenever suitable.*
☒	☐	For Bayesian analysis, information on the choice of priors and Markov chain Monte Carlo settings
☒	☐	For hierarchical and complex designs, identification of the appropriate level for tests and full reporting of outcomes
☒	☐	Estimates of effect sizes (e.g. Cohen's d, Pearson's r), indicating how they were calculated

Our web collection on statistics for biologists contains articles on many of the points above.

Software and code

Policy information about availability of computer code

Data collection	EXCEL was used for data collection.
Data analysis	Graphpad Prism 8.4.0 and jvenn (jvenn.toulouse.inra.fr) were used to plot figures and venn diagram. SPSS 24.0 was used for statistical analyses.

For manuscripts utilizing custom algorithms or software that are central to the research but not yet described in published literature, software must be made available to editors and reviewers. We strongly encourage code deposition in a community repository (e.g. GitHub). See the Nature Research guidelines for submitting code & software for further information.

Data

Policy information about availability of data

All manuscripts must include a data availability statement. This statement should provide the following information, where applicable:

- Accession codes, unique identifiers, or web links for publicly available datasets
- A list of figures that have associated raw data
- A description of any restrictions on data availability

Source data are provided with this paper.

Field-specific reporting

Life sciences study design

All studies must disclose on these points even when the disclosure is negative.

Sample size	In this observational study, 1006 eligible vaccine recipients were enrolled for safety assessments. To asses the immunogenicity of the vaccine, 760 participants had specific antibody immunoassay and neutralization assay on day 28 after the second dose. When 700 participants are enrolled, we have 80% power to demonstrate success on the primary efficacy hypothesis that the seroconversion rate of virus specific antibody being greater than 96%, when assuming the true seroconversion rate is 98%, with a 10% dropout rate based on a one-sided exact test with significance level (alpha) of 0.025 in PASS13. The sample size of 760 participants is larger than 700, which is sufficient to provide >80% power.
Data exclusions	No data was excluded in this study.
Replication	Samples were analyzed in duplication for neutralization assay. All attempts at replication were successful. The averaged results from the duplication were reported in this study. Samples were tested once for chemiluminescent immunoassay and cytokine measurement.
Randomization	This is a prospective observational study, where randomization is not relevant to this study.
Blinding	This is an observational non-randomized study, therefore blinding is not applicable. Samples were detected blinded for neutralization assay.

Reporting for specific materials, systems and methods

We require information from authors about some types of materials, experimental systems and methods used in many studies. Here, indicate whether each material, system or method listed is relevant to your study. If you are not sure if a list item applies to your research, read the appropriate section before selecting a response.

Materials & experimental systems

n/a	Involved in the study
☒	☐ Antibodies
☐	☒ Eukaryotic cell lines
☒	☐ Palaeontology and archaeology
☒	☐ Animals and other organisms
☐	☒ Human research participants
☐	☒ Clinical data
☒	☐ Dual use research of concern

Methods

n/a	Involved in the study
☒	☐ ChIP-seq
☒	☐ Flow cytometry
☒	☐ MRI-based neuroimaging

Eukaryotic cell lines

Policy information about cell lines

Cell line source(s)	HEK-293T cells were obtained from ATCC.
Authentication	The cell lines were not authenticated.
Mycoplasma contamination	All cell lines were tested negative for mycoplasma.
Commonly misidentified lines (See ICLAC register)	Not commonly misidentified cell lines were used in the study.

Human research participants

Policy information about studies involving human research participants

Population characteristics	Among 1006 vaccine recipients, 284 were male and 722 were female, with a median age of 35.00 (28.00-43.00) years.
Recruitment	From January 14, 2021 to March 10, 2021, healthcare workers in Shanghai Ruijin Hospital, aged 18-59 years, with negative serum specific antibodies against SARS-CoV-2 at the time of screening (V1), and willing to receive two doses, 21 days apart of inactivated SARS-CoV-2 vaccine (BBIBP-CorV, Sinopharm) were eligible participants and were recruited in this study. Blood samples tested for neutralization assay were taken from the enrolled participants on a voluntary basis without any selection. Under these conditions, there is no particular bias.
Ethics oversight	Written informed consent was obtained from all participants before the screening. The protocol and informed consent were approved by the Ethics Committee of Shanghai Ruijin Hospital (RJHKY2021-12) in accordance with the Declaration of Helsinki and Good Clinical Practice.

Note that full information on the approval of the study protocol must also be provided in the manuscript.

Clinical data

Policy information about clinical studies

All manuscripts should comply with the ICMJE guidelines for publication of clinical research and a completed CONSORT checklist must be included with all submissions.

Clinical trial registration	This study is registered with ClinicalTrials.gov, NCT04795414.
Study protocol	The study protocol can be accessed in the supplementary information file.
Data collection	All relevant clinical data of enrolled participants was collected from the Ruijin Hospital electronic system. The recruitment time period is from January 14, 2021 to March 10, 2021. Data collection was completed as of August 30, 2021.
Outcomes	The primary safety endpoint was any adverse reactions within 28 days after each dose of vaccination. The secondary safety endpoint was any clinical laboratory abnormalities within 28 days after each dose of vaccination. The primary immunogenic endpoints were the seroconversion rate and the titers of specific antibodies and neutralizing antibodies against SARS-CoV-2 on day 28 post the second dose, and the secondary immunogenic endpoints were the seroconversion rate and the titers of specific antibodies and neutralizing antibodies against SARS-CoV-2 on day 180 post the second dose.
